# 3D Printing with Tragacanth-Gum-Based Bioinks: A New Frontier in Bioprinting Materials

**DOI:** 10.3390/gels12020152

**Published:** 2026-02-07

**Authors:** Shivani Dogra, Bhupendra Koul, Ananta Prasad Arukha, Muhammad Fazle Rabbee

**Affiliations:** 1 Department of Microbiology, School of Bioengineering and Biosciences, Lovely Professional University, Phagwara 144411, India; shivanidogra241995@gmail.com; 2 Department of Biotechnology, School of Bioengineering and Biosciences, Lovely Professional University, Phagwara 144411, India; 3 Department of Infectious Disease and Immunology, College of Veterinary Medicine, University of Florida, Gainesville, FL 32110, USA; ananta.arukhaa@gmail.com; 4 Department of Biotechnology, Yeungnam University, Gyeongsan 38541, Republic of Korea

**Keywords:** tragacanth gum, bioinks, extrusion bioprinting, hydrogel scaffolds, tissue engineering, biocompatibility

## Abstract

Extrusion-based bioprinting is widely used for fabricating cell-laden constructs; however, its success is highly dependent on the rheological and biological performance of the bioink. Natural polysaccharide gums have emerged as promising bioink components due to their biocompatibility and tunable properties. Among them, tragacanth gum (TG), a complex anionic heteropolysaccharide composed of tragacanthin and bassorin fractions, has gained increasing attention for extrusion bioprinting applications. TG exhibits pronounced shear-thinning behavior, high water uptake, and spontaneous gel-forming ability, which collectively enhance the printability, shape fidelity, and structural stability of bioinks. This review critically summarizes recent advances in TG-based hydrogels and bioinks, with emphasis on their molecular characteristics, rheological and physicochemical properties, and biological performance in extrusion bioprinting systems. The role of TG as a functional component in composite bioinks, particularly in improving mechanical integrity, extrusion consistency, and cytocompatibility, is discussed. Finally, current challenges and future research directions are highlighted to support the development and clinical translation of TG-based bioinks for tissue engineering applications.

## 1. Introduction

Extrusion-based 3D bioprinting has emerged as a powerful technique in tissue engineering and regenerative medicine [1,2]. It allows researchers to fabricate 3D cell embedded structures with spatial control over both materials and cell placement during fabrication. In extrusion printing, bioinks that are typically hydrogel systems may or may not be loaded with cells that are extruded through a nozzle using pneumatic or mechanical force [3]. The printed filaments are deposited layer by layer, resulting in the formation of stable 3D constructs in a controlled manner [4]. For a material to function as an effective bioink, it must meet essential requirements such as printability, mechanical properties, cytocompatibility, and rheological and degradation behavior that must support tissue formation [5].

For a formulation to perform well as a bioink, the materials should support both printing and cell viability. Excessive shear forces generated within the nozzle can damage embedded cells. Therefore, bioinks need shear protection rheological characteristics that minimize shear stress during printing [6]. Ideally, the material should remain sufficiently fluid to reduce shear stress during extrusion or be followed by a sufficient increase in gelation or viscosity upon deposition to stabilize the printed structure [7].

During extrusion, the printable material/ink should flow uniformly under applied pressure, but retain shape fidelity once deposited. This requires properties such as rapid recovery of viscosity post extrusion, shear-thinning behavior, and an adequate yield stress that enables the formation of continuous filaments and stacked layers without collapse [8,9,10,11].

When crosslinking or gelation is required, the bioink must possess gel-forming potential to form a stable hydrogel, which should be compatible with cell viability. Following printing, the hydrogel should provide adjustable mechanical features such as tailored elasticity and stiffness along with controllable degradation behavior. The material can match the requirements of the biomechanical profile of regenerating tissue and support cell-mediated remodeling. Together, these combined requirements—crosslinking, remodeling, cytocompatibility, and degradation behavior—make bioink design a highly interdisciplinary and technically challenging task [12,13]. In the context of these criteria, selecting an appropriate base polymer is critical. Polysaccharides derived from seeds, plants, trees or other natural sources are promising substitutes for synthetic polymers. They offer multiple benefits like being widely available, renewable, non-toxic, biodegradable and relatively inexpensive. From a chemical perspective, they carry functional groups (-OH, -COOH) that can enable both physical gelation and chemical crosslinking, while their composition often resembles a native extracellular matrix, promoting cell adhesion, viability and function [14].

**Figure 1:**
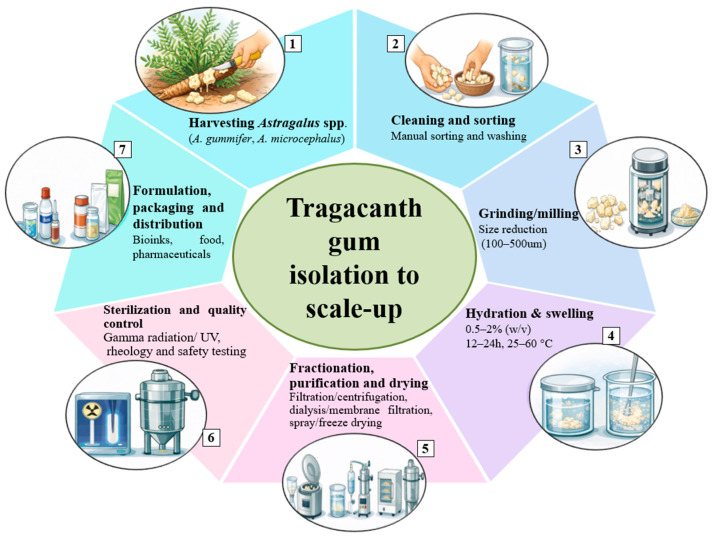
Schematic representation of the isolation, purification, and packaging of tragacanth gum (TG) from *Astragalus* species.

Among various natural gums, TG has gained attention due to its natural origin and various properties. Figure 1 presents a schematic overview of the isolation, purification and scale-up process of TG from *Astragalus* species, outlining the key steps involved from raw exudate collection to commercial scale applications.

**Figure 2:**
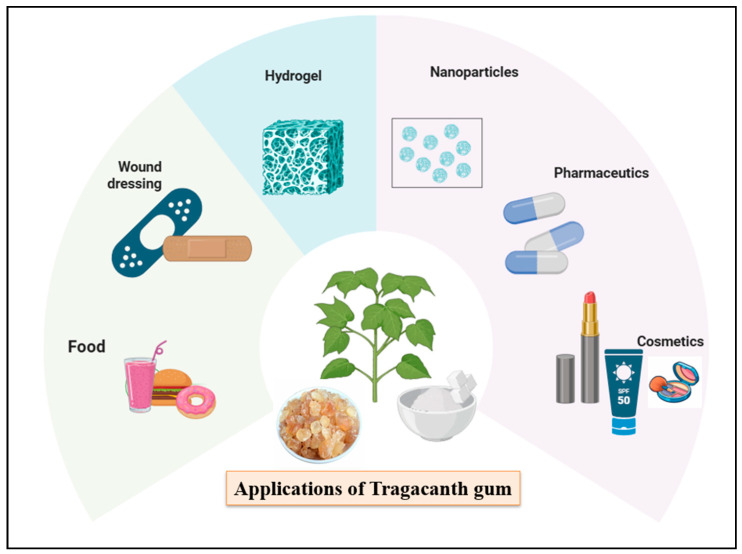
Applications of tragacanth gum across biomedical and technological fields.

It is an anionic heteropolysaccharide with water-soluble and water-swelling fractions. These offer gelation, high swelling, biocompatibility, biodegradability, shear thinning behavior and regulatory acceptance, making it an excellent candidate for extrusion-based bioinks [15]. It has long history of safe use in the food, cosmetic and pharmaceutical fields, as represented in Figure 2. This sets an example of biodegradability, biocompatibility, minimal toxicity, stability over varied pH, and temperature and regulatory acceptance as a food additive or pharmaceutical excipient [16,17,18].

Due to these properties, there has been growing interest in biomaterials and tissue engineering [2,19]. TG-based hydrogels have been explored for wound healing, drug delivery, and bone and soft tissue engineering [20,21]. They have been reported to be non-carcinogenic, non-toxic and biodegradable, which highlights their potential as ECM-mimicking scaffolds. There is contemporary work that investigates the use of TG alone or in composites to improve the rheological performance and printability of bioprinting inks [22]. In this context, the present review aims to provide a comprehensive overview of the capabilities of TG-based bioinks for extrusion-based 3D bioprinting. Section 1 summarizes the essential requirements for bioinks in extrusion bioprinting, followed by discussion on natural polymers and TG as a promising candidate. Subsequent sections cover TG’s historical industrial usage, chemical composition, rheological and physicochemical behavior, and biodegradability and biocompatibility. It is followed by state-of-the-art research including composites and explored TG-based hydrogels and bioinks for tissue engineering and bioprinting. This review concludes by highlighting present limitations and recommendations for the future development of TG-based bioink(s) technology.

## 2. Chemistry, Structure and Crosslinking of Tragacanth Gum

Tragacanth gum is usually obtained from wounded roots and stems of several *Astragalus* species, with *A. gummifer*, *A. brachycalyx*, *A. adscendens*, and *A. tragacantha* known to be the most common commercial sources [23,24,25,26]. The major producers of TG are Turkey, Iran and nearby regions. It is traditionally marketed in different forms, such as high-grade “ribbons”, flakes (kharmony), granules and powders. Historically, Iranian “ribbon” grades are of superior quality among several commercial grades [27,28,29]. TG is structural complex anionic mixture consisting of two major fractions: (1) Tragacanthin: A highly water-soluble fraction composed of branched type-II arabinogalactan (with galactose and arabinose backbone/side chains) that behaves like a random coil conformation when dissolved in solution. (2) Bassorin: Water-swellable and partially soluble in water, that hydrates to form a dense gel network. This fraction is responsible for the strong gelation and viscoelastic behavior of the native gum.

**Figure 3:**
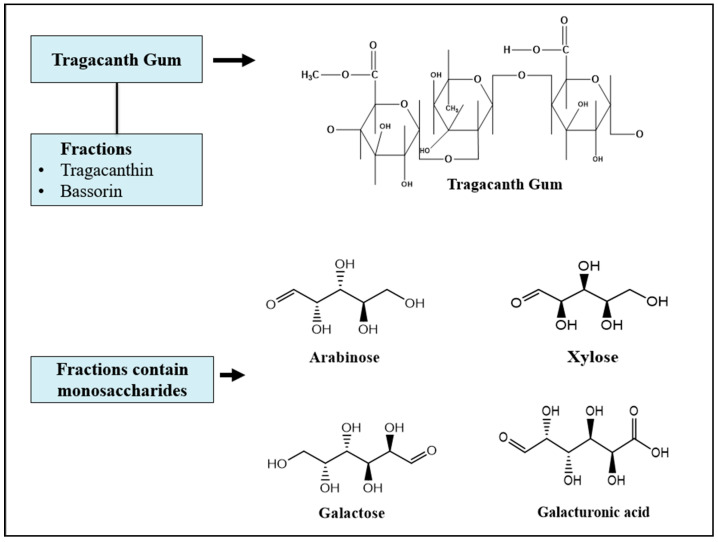
Chemical structure of tragacanth gum and its fractions containing monosaccharides.

Both fractions contain several monosaccharides, including D-galacturonic acid, D-galactose, L-arabinose and D-xylose, and associated cations Ca^2+^, Mg^2+^ and K^+^ [30] as represented in Figure 3. The main reactive functional groups available for modification are the hydroxyl groups on sugar rings and carboxyl/uronic acid groups derived from galacturonic acid and other acidic residues. These functional groups enable esterification, etherification, amidation and radical grafting. The ratio of tragacanthin to bassorin among species and commercial grades account for batch-to-batch variability in solubility and rheology [31,32].

### 2.1. Crosslinking Mechanisms of Tragacanth Gum Based Hydrogels

The crosslinking mechanisms of gum-based hydrogels include physical crosslinking (ionic and polyelectrolyte complexation), chemical (covalent) crosslinking, enzymatic and mild crosslinking, and hybrid strategies (ionic + covalent) [33,34,35].

#### 2.1.1. Physical Crosslinking

TG is an anionic polysaccharide, which forms gels through ionic interactions with divalent cations like Ca^2+^. Additionally, polyelectrolyte complexation between TG and cationic polymers like chitosan has been reported [36]. These interactions create reversible networks that stabilize the hydrogel structure without covalent bonding, allowing easy processing and mild gelation. Such physical gels are commonly used in drug delivery and biofabrication [37,38].

#### 2.1.2. Chemical Crosslinking

Covalent crosslinking includes carboxymethylation followed by glutaraldehyde crosslinking, graft polymerization, and methacrylation followed by photopolymerization. Carboxymethylation is carried out by treating TG with monochloroacetic acid under alkaline conditions. This replaces some hydroxyl groups with carboxymethyl units (–CH_2_–COO^−^). This chemical modification increases the anionic character of the polymer and promotes hydration and dispersion in water. A higher degree of substitution results in better solubility and makes gums more chemically reactive for subsequent blending or crosslinking with other biopolymers. Although, this modification can reduce the high viscosity seen in native TG gels. Carboxymethylated tragacanth gum (CMT) is often used to prepare drug delivery systems and as an intermediate for bioink and hydrogel preparation. Methacrylation involves the incorporation of methacrylate groups into the TG using methacrylic anhydride or glycidyl methacrylate reagents. The resulting vinyl groups make the modified polymer responsive to photopolymerization, which further forms covalently crosslinked networks upon exposure to UV or radical generated conditions. It is widely used to convert natural polymers into printable photo-curable bioinks [39]. Graft polymerization is also known as the radical grafting of synthetic monomers. It introduces free radical sites onto the polymer backbone to grow synthetic polymer chains, such as polyacrylamide, polyacrylic or related derivatives. It enhances the strength, swelling capacity or controlled drug release behavior. These are widely explored for wound dressings, hydrogels, adsorbents and functional coatings [40,41,42].

#### 2.1.3. Enzymatic and Mild Crosslinking

Enzyme-mediated approaches, such as horseradish peroxidase (HRP) with H_2_O_2_, can crosslink polysaccharides that contain phenolic or similar reactive groups. This method allows gentle gelation compatible with live cells and supports tunable reaction kinetics. Mild irradiation methods (e.g., electron beam) have also been used to crosslink TG composites with polymers like PVA, enabling applications in tissue engineering [43].

#### 2.1.4. Hybrid Strategies

Hybrid crosslinking uses a fast ionic network to preserve the filament shape during printing with a secondary covalent curing step to enhance long-term strength and mechanical reinforcement. This approach enables the printed structures to maintain gel stiffness, shape fidelity and controlled degradation profiles in the engineered tissue [44,45]. The extent of hydrogel crosslinking is generally evaluated using analytical methods including gelation kinetics monitored by in situ rheological measurements, swelling ratio analysis, and water absorption analysis. Mechanical performance is often characterized by compression tests, dynamic mechanical analysis, and the estimation of network mesh size. In addition, degradation behavior is evaluated through enzymatic or hydrolytic breakdown studies. Collectively, these tests determine the hydrogel stability, network structure and overall suitability for biomedical applications.

## 3. Rheology for Extrusion Printing

Extrusion-based bioprinting is highly influenced by the rheological behavior of the bioink. An ideal bioink exhibits shear-thinning behavior, as its viscosity decreases under applied shear stress for smooth extrusion through the nozzle, but remains highly viscous on deposition to maintain the filament shape [4,8,46]. To prevent the filament spreading or its structure collapsing after deposition, the bioink must also possess an adequate yield stress. Additionally, thixotropy recovery is also important, which is the capacity to regain viscosity after extrusion, as it ensures that adjacent layers maintain their structure and do not merge. During the printing process, the viscoelastic behavior with the storage modulus (G′) exceeds the loss modulus (G″) and enables the hydrogel to behave like a soft solid [47,48]. Temperature sensitive rheology is to be carefully controlled for inks based on thermoresponsive materials, so as to maintain appropriate viscosity under physiological conditions, and for the temperature-sensitive rheology to be carefully controlled [49,50,51].

Key rheological parameters including storage modulus (G′), loss modulus (G″), yield stress, and viscosity are routinely employed to assess printability under relevant processing conditions. In blended TG-based hydrogels incorporating cellulose nanocrystals, a viscoelastic profile characterized by dominant elastic behavior (G′ > G″) across applicable frequency ranges has been reported, indicating solid-like stability at rest while retaining sufficient flow under shear, a prerequisite for uniform filament deposition [22].

Quantitative correlations between rheological properties and printability have also been established. Elevated storage modulus and yield stress contribute to filament integrity, resistance to collapse, and reliable layer-by-layer stacking, whereas controlled viscosity under shear promotes continuous extrusion without nozzle blockage. Collectively, optimization of these rheological parameters has been shown to improve shape fidelity and printing resolution while reducing shear-induced cellular damage during the bioprinting process [52].

### 3.1. Rheology of TG and TG-Based Hydrogels

Due to the highly branched polysaccharide structure, TG forms viscous and pseudoplastic solutions, even at relatively low concentrations [53,54]. The concentration of polymer strongly affects viscosity and yield stress, with a critical threshold necessary to ensure continuous and structurally stable filament formation during printing. Chemical modifications such as carboxymethylation can further improve the mechanical performance of hydrogels by increasing gel strength, enhancing the storage modulus and promoting chain hydration. Blending TG with other polymers or nanoscale fillers such as alginate, gelation or cellulose nanocrystals [55,56,57] contributes to improved network stability, viscoelastic behavior, and overall shape fidelity of printed constructs. Collectively these characteristics make TG-based hydrogels strong candidates for extrusion printing applications [20,58,59].

### 3.2. Rheology Measurement and Printable Metrics

Bioink rheology is commonly assessed using a combination of rotational and oscillatory measurements. The rotational flow curves are used to determine the shear-thinning behavior, whereas oscillatory tests measure the storage modulus (G′) and loss modulus (G″). Yield stress is generally quantified through stress ramp protocols or from flow curve analysis. Thixotropic properties are examined using recovery tests that are performed to simulate nozzle extrusion and the ability of the material to regain viscosity [50,60,61].

Rheological properties are critical for assessing the printability of tragacanth gum (TG)-based bioinks, as successful extrusion requires a balance between flow under shear and post-deposition stability. TG forms physically stabilized networks via hydrogen bonding and chain entanglement, resulting in pronounced shear-thinning and rapid viscosity recovery. TG-based and TG-blended hydrogels, including TG/cellulose nanocrystal composites, exhibit non-Newtonian behavior with G′ exceeding G″, supporting stable filament formation, shape fidelity, and moderate extrusion pressures [22,58]. Acting primarily as a rheology modifier rather than a load-bearing polymer, TG enhances zero-shear viscosity and yield stress even at low concentrations (0.5–2 wt%), reducing shear stress on encapsulated cells compared with alginate, and providing more flexible, reproducible printing than thermoresponsive gelatin-based bioinks [62]. Overall, TG improves extrusion performance, filament uniformity, and post-printing stability, highlighting its utility as a functional additive in composite bioinks.

### 3.3. Extrusion Bioprinting Process

As mentioned before, extrusion bioprinting is a commonly employed biofabrication approach for producing three-dimensional, cell-containing constructs through the sequential deposition of continuous bioink filaments. In this technique, bioinks incorporating living cells and/or bioactive molecules are dispensed through a nozzle or micro-needle under precisely regulated parameters such as pressure, extrusion speed, and temperature to generate constructs with defined geometries. The printing trajectory is guided by computer-aided design (CAD) models, enabling accurate spatial placement of materials and facilitating the recreation of complex tissue-like architectures. Extrusion-based systems may operate using pneumatic, mechanical, or piston-driven mechanisms, each providing varying degrees of control over flow behavior and shear forces; therefore, careful optimization of these parameters is essential to preserve cell viability during the printing process [63].

## 4. Cell Viability and Biological Performance

During extrusion bioprinting, embedded cells experience mechanical stress that can influence their biological function and viability. Parameters such as extrusion pressure, nozzle size, shear forces within the nozzle, and the nature and concentration of crosslinking agents may cause cytotoxic effects if not carefully controlled. Optimizing printing parameters together with tailoring bioink rheology can reduce the cellular stress while still maintaining structural fidelity [64,65]. TG-based hydrogels, when formulated with other biopolymers, have demonstrated the ability to maintain cell viability post printing, and support subsequent proliferation. Research indicates that TG-containing bioinks allow continuous cell growth and in vitro functional performance, including scaffold-based assays and wound-healing models. Such bioinks often perform comparably or better than alginate-based controls.

Studies evaluating tragacanth gum (TG)-based hydrogels and bioinks commonly report sustained and progressive cell proliferation over culture periods of 3–14 days, rather than a single absolute proliferation rate, with typical increases of approximately 1.3–2.0-fold in metabolic activity over 7 days [22,58]. Compared to other natural polymers, TG-containing systems generally demonstrate higher proliferation than pure alginate hydrogels, which lack intrinsic cell-adhesive motifs unless functionalized, and show proliferation levels comparable to gelatin or GelMA-based matrices. Gelatin-based systems may support more rapid initial cell adhesion due to integrin-binding sites [22,66]. Compared to chitosan, TG-based gels frequently exhibit equal or improved proliferation under physiological pH conditions, where unmodified chitosan may limit cell growth [67]. Overall, these findings indicate that TG is not inferior to widely used biopolymers and, particularly in blended formulations, supports continuous cell proliferation and functional performance by improving hydrogel hydration, pore interconnectivity, and mechanical stability [58,68].

Several strategies have been employed to improve cell viability during extrusion bioprinting. The shear-thinning nature of TG-based bioinks helps reduce nozzle-induced shear stress, while gentle crosslinking methods such as ionic or mild chemical crosslinking are preferred over harsh treatments to preserve cell integrity [22,58]. Incorporating cytoprotective additives, including proteins, sugars, or polyethylene glycol (PEG), can further mitigate mechanical damage to cells during extrusion. Co-printing with sacrificial materials has also been shown to provide temporary protective barriers around encapsulated cells, enhancing survival and uniform distribution [62]. Beyond cellular protection, life-cycle assessments (LCA) highlight the ecological advantages of TG as a plant-derived natural polymer. Compared to synthetic bioinks, TG offers biodegradability, low-energy production, and sustainable sourcing, while maintaining high printability and cytocompatibility biomanufacturing [20,68].

Hydrogels formulated solely with tragacanth gum (TG) demonstrate consistently high cytocompatibility, with 85–95% cell viability in fibroblast, osteoblast-like, and epithelial cell lines, depending on the formulation concentration and crosslinking parameters. Notably, blending TG with other natural or synthetic polymers such as alginate, gelatin, cellulose derivatives, chitosan, or polyvinyl alcohol (PVA) does not compromise biocompatibility; instead, cell viability is often preserved or further improved, frequently surpassing 90–100% in standard in vitro evaluations, including MTT and Live/Dead assays. Several investigations also highlight enhanced cellular proliferation in composite hydrogel systems relative to TG alone, which is primarily attributed to improved mechanical integrity, optimized pore architecture, and strengthened cell–matrix interactions.

Post-printing cell viability is strongly governed by both bioink rheological properties and extrusion-related processing conditions. Cells are exposed to shear stresses during extrusion that may induce deformation or loss of viability; experimental and theoretical studies consistently demonstrate that elevated shear stress arising from higher bioink viscosity, reduced nozzle diameters, or increased extrusion rates can adversely affect cell survival. Conversely, appropriate optimization of these parameters significantly improves cell viability during and after printing [63].

In the case of tragacanth gum (TG)-based bioinks, recent investigations report that composite formulations incorporating TG with nanocellulose or other polymeric components achieve favorable rheological characteristics for extrusion while simultaneously supporting high levels of cell viability and proliferation within three-dimensional constructs. These TG-containing scaffolds have been shown to exhibit non-cytotoxic behavior and enhanced cellular metabolic activity across multiple cell types, which is largely attributed to their highly hydrated nature and porous network that promotes efficient nutrient and waste transport [20].

Furthermore, the pronounced swelling behavior and interconnected macroporous architecture observed in TG/CNC systems facilitate uniform cell distribution and effective mass transfer throughout printed scaffolds. Such structural features are closely associated with improved cell survival and the potential development of tissue-like functionality in bioprinted constructs [22].

### Comparison of Tragacanth Gum with Alginate, Gelatin and Pectin Based Bioinks

Among hydrogels, alginate (via fast iconic Ca^2+^) and GelMA (via fast photo crosslinking) are considered the most reliable locking mechanisms after deposition commonly used for extrusion-based bioprinting. Pectin is also capable of forming hydrogel networks through either iconic or chemical crosslinked routes. TG hydrogels usually need additional polymer for blending or chemical modification, such as methacrylation, or the addition of Ca^2+^ to achieve quick gelation and high structural stability during printing [69]. When alginate and GelMA are formulated, it supports smooth filament formation and stable layer-by-layer stacking. TG alone shows highly shear-thinning behavior, with high swelling, i.e., it has good filament shape but needs optimization per formulation. For this reason, many studies use TG as a rheology modifying component by incorporating it into an alginate- or gelatin-based system to improve print resolution, viscosity control, and mechanical strength [70]. In terms of cell compatibility, both GelMA and optimized alginate formulations frequently support >80% short term cell viability. TG blends show good cytocompatibility, but available literature suggests long-term cell biological interactions and matrix-mediated cell responses are not extensively characterized as alginate or GelMA. Table 1 provides a comparison of the key parameters for TG, alginate/sodium alginate, gelatin/GelMA, and pectin. 

## 5. Formulation Design and Processing for Bioinks

Pure TG bioinks are generally formulated at low to moderate polymer concentrations, often in the range of 1–4% *w*/*v* [62,77,78]. However, pure TG exhibits high swelling and limited mechanical stability, which can reduce the structural fidelity of printed constructs. To overcome these limitations, TG is frequently blended with other biopolymers including gelatin, alginate and pectin. These combinations enhance mechanical strength, improve gelation behavior and enable more controllable rheology. Reported formulations include TG/gelatin mixtures (2–3% TG + 5–10% gelatin) for soft tissue scaffolds, as well as TG/alginate blends (1–3% TG + 2–5% alginate) used in bone tissue engineering. These blends support shear thinning behavior and viscosity adjustment, and provide improved stability post printing [62,79,80].

### 5.1. Additives to Enhance Printability and Functionality

In addition to polymer blending, the performance of TG-based bioinks can be improved by adding nano- or micro-scale additives. Materials such as nanoclays, CNCs, graphene oxide and various bioactive nanoparticles are used to boost the mechanical strength and shear thinning, and promote bioactivity. For instance, combining CNCs with TG has been shown to increase the storage modulus, maintain the printed scaffold structure, and support more stable filament formation over long-term cell culture in the hydrogel [70]. Such reinforcement approaches are especially valuable for multi-layered constructs or designs that require high dimensional accuracy [80,81,82]. TG-based bioinks are typically extruded through nozzles ranging from 200 to 410 µm at pressures of 20–80 kPa and printing speeds of 5–20 mm/s, supporting cell densities of 1 × 10^6^–5 × 10^6^ cells/mL without compromising printability or viability [58,62]. Their pronounced shear-thinning behavior and high water content allow stable filament formation at moderate pressures, reducing shear-induced cell damage, while rapid post-extrusion viscosity recovery preserves structural integrity. Compared to other natural bioinks, TG blends are advantageous, as alginate often requires higher pressures or RGD functionalization for effective cell adhesion; gelatin/GelMA is temperature-sensitive, and chitosan can be unstable at neutral pH.

### 5.2. Cell-Laden Bioink Processing Parameters

The performance of cell-laden-based bioinks is influenced by the printing parameters applied during fabrication. The cell density must be chosen carefully to balance biological function without making the ink too viscous or difficult to extrude. Parameters such as nozzle size, extrusion pressure, printing speed, and temperature control play a major role in reducing shear-related damage and maintaining high cell viability. Sterilization methods such as UV exposure, filtration or other compatible methods must be suitable for both cells and polymer components. When these parameters are properly optimized, the printed constructs retain their shape while supporting cell survival and proliferation [83,84]. Bioinks must be handled and prepared under sterile conditions. They are typically stored at 4 °C to prevent microbial growth and to maintain their intended rheological properties. Before use, pre-formulated bioinks may require gentle stirring to restore homogeneous viscosity. When long-term storage is needed, freezing or lyophilization can be used, provided that the rehydration process maintains both polymer structural integrity and cellular compatibility. Overall, proper storage and handling are essential to ensure consistent scaffold quality and achieve reproducible bioprinting outcomes [85,86,87]. TG-based bioinks should be prepared under sterile conditions, and standard sterilization methods such as filtration or UV treatment can be used without affecting rheological properties [20,58]. They are stable at 4 °C, and gentle stirring before use restores homogeneity. Long-term storage via freezing or lyophilization is possible, provided rehydration maintains polymer integrity and cytocompatibility. Unlike thermoresponsive bioinks, TG does not require strict temperature control, simplifies handling compared with alginate or GelMA, and enhances reproducibility.

Tragacanth gum (TG)-based and TG-composite bioinks are commonly extruded using nozzle diameters in the range of 200–410 µm, with applied pressures of approximately 20–80 kPa and printing speeds of 5–20 mm s^−1^, while consistently maintaining post-printing cell viabilities above 85–90% [58]. These operational parameters closely align with those reported for other widely used natural bioinks, including alginate, gelatin, and chitosan. Notably, TG-containing formulations often require only moderate extrusion pressures, which can be attributed to their pronounced shear-thinning behavior and high water content, factors that collectively help mitigate shear-induced cellular damage during printing [62].

Regarding cellular loading, TG-based bioinks typically accommodate cell densities ranging from 1 × 10^6^ to 5 × 10^6^ cells mL^−1^, comparable to gelatin- and alginate-based systems, without negatively impacting printability or cell survival. In contrast to thermoresponsive bioinks such as gelatin, temperature regulation is generally less critical for TG formulations, as gel formation primarily depends on physical chain entanglement and ionic or chemical crosslinking mechanisms rather than thermal gelation. Furthermore, conventional sterilization strategies, including precursor solution filtration and ultraviolet treatment, have been effectively applied to TG bioinks without compromising their rheological behavior or cytocompatibility, consistent with observations for other polysaccharide-based materials.

### 5.3. Formulation Strategies to Tune TG Rheology

TG-based bioinks can be systematically engineered by incorporation of or blending with polymers or nanofillers [88]. Blending TG with ionic polymers such as alginate increases network rigidity and the elasticity of the hydrogel network, whereas gelatin provides thermo-responsive viscosity and promotes cellular adhesion. The cellulose nanocrystals reinforce the network to enhance shear-thinning and thixotropic recovery. Synthetic additives like PEG or PVA can further tune dehydration and viscoelasticity properties. Together, these strategies contribute to a higher elastic modulus, improve filament formation, and improve structural fidelity during extrusion printing. This enables TG-based hydrogels to be applied in tissue engineering applications. Table 2 compiles peer-reviewed experimental and review studies published from the last 10 years that explore TG either as a primary hydrogel matrix or as a rheological modifier in composite bioinks.

## 6. Applications

TG-based bioinks exhibit excellent water retention, adjustable rheology, and cytocompatibility [15,94,95]. This makes it possible to create stable and cell-supporting structures. They show promising results in wound healing and tissue regeneration where bioactivity and hydration promote tissue repair. TG provides controlled release and enhances the print quality in biofabrication and drug delivery [96].

Tragacanth gum (TG) should be regarded not as a primary bioink matrix but as a multifunctional supporting component that significantly improves extrusion bioprinting performance. Owing to its ability to form physically stabilized networks through hydrogen bonding and chain entanglement, TG contributes to network stabilization, enhances ionic or secondary crosslinking efficiency, and allows further tunability when chemically modified. Its principal contribution lies in rheological control, where TG imparts shear-thinning behavior, thixotropic recovery, and improved viscosity, thereby enhancing print fidelity, filament stability, and pore preservation. Although TG itself exhibits limited intrinsic bioactivity, its high biocompatibility and hydration capacity indirectly promote favorable biological outcomes by supporting cell viability, nutrient diffusion, and structural consistency in composite systems. Consequently, TG is best utilized as a functional bioink additive, with future designs focusing on hybrid crosslinking strategies, chemical modification, and tissue-specific optimization to fully exploit its supportive role in bioprinting applications [20,22,89,90,91,92,93]

### 6.1. Tissue Engineering

TG-derived bioinks have shown significant potential in fabricating tissue-engineered constructs such as skin, bone, tissue and vascular grafts [97,98]. TG hydrogels, when blended with materials like collagen and polyurethane, exhibit remarkable swelling capacity, anti-microbial activity and enhanced fibrillogenesis, supporting efficient wound and tissue repair [99]. Overall, TG has the ability to form stable, cell-supportive scaffolds and is suitable for fabricating complex tissue constructs. In tissue engineering, TG is recognized as a biocompatible and biodegradable polysaccharide that has been successfully utilized in wound healing and scaffold design, where it supports cell adhesion, proliferation, and extracellular matrix development. TG-based hydrogels developed for wound-dressing applications exhibit high swelling capacity, controlled drug release, and excellent compatibility with skin-related cells, leading to improved moisture retention and accelerated repair when evaluated through cell viability, migration, and histological analyses [20]. Moreover, hybrid TG scaffolds incorporating polymers such as chitosan, collagen, or polyurethane demonstrate enhanced mechanical stability, antibacterial activity, and fibroblast proliferation, all of which are critical for skin and soft-tissue regeneration [99]. Although direct applications of TG in vascular graft analogs remain limited, its hydrophilic and porous matrix supports cell infiltration and morphology, and recent nanocomposite TG systems developed for related biomedical uses further emphasize its potential for future vascular tissue engineering strategies [68].

### 6.2. Wound Healing and Dressings

TG-based hydrogels have been attractive for wound care research due to their bioactive properties, biocompatibility and ability to retain moisture. When combined with a natural extract such as *Artemisia vestita*, which is known to be a rich source of bioactive constituents [100], they have demonstrated strong in vitro performance and enhanced wound closure, and promoted tissue regeneration [101]. Another study reported a TG crosslinked hydrogel with good biocompatibility, strong swelling and effective vancomycin delivery for wound healing use [102], while another physically crosslinked PVA/chitosan/TG hydrogel with added vitamin E enhanced antioxidant activity and also supported the wound repair mechanism [103].

### 6.3. Drug Delivery and Controlled Release

TG hydrogels are recognized as efficient carriers for controlled drug delivery. Their adjustable swelling behavior and polymer network enable the sustained release of therapeutic drugs within tissue engineering applications [20]. When incorporated into bioinks, TG can support both drug encapsulation and structural formation of the printed scaffold. This results in tissue support with drug-releasing capability. TG-based hydrogel carriers have been extensively investigated for their capacity to modulate drug loading and release behavior through variations in polymer composition, crosslinking density, swelling properties, and environmental responsiveness. Recent studies demonstrate that TG-containing nanohydrogels can achieve pH- and temperature-responsive, diffusion-controlled drug release, with tunable kinetics governed by the polymeric network structure [96]. Chemically modified TG systems, such as carboxymethylated derivatives, as well as TG-based wound-dressing hydrogels loaded with anti-inflammatory agents or antibiotics, exhibit sustained release profiles, mechanical robustness, and antimicrobial efficacy, underscoring their suitability for topical and localized therapeutic applications [104]. Overall, these findings clarify how physicochemical parameters including crosslinking degree, blending ratio, and swelling behavior govern release mechanisms in TG-based systems, highlighting TG as a versatile natural polymer compared to established carriers such as alginate, chitosan, and gelatin.

### 6.4. Biofabrication

Advances in natural-gum-based bioprinting extend well beyond single material scaffolds [105]. These bioinks have been used in complex biofabrication methods including sacrificial ink approaches and multi-material printing. This allows the creation of constructs with intricate architectures [106], functional gradients and multi-level porosity [107]. Such approaches are essential for mimicking the mechanical and biological complexity of natural tissues [108]. This demonstrates the remarkable flexibility of polymers in next-generation 3D bioprinting applications [109].

**Figure 4:**
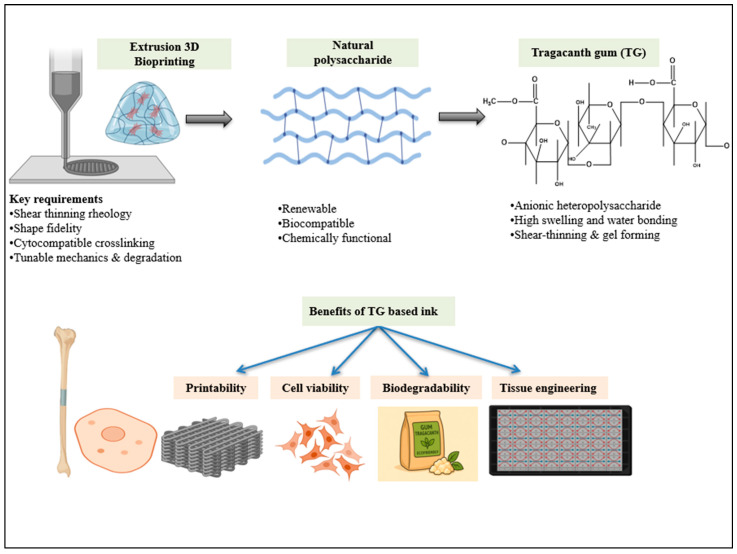
Overview of tragacanth-gum-based bioinks for extrusion bioprinting.

Figure 4 outlines the design and application landscape of TG-based bioinks in extrusion bioprinting, structural formulation, crosslinking methods, and printing performance, and their translation into tissue engineering applications.

## 7. Research Gaps and Future Perspectives

TG has several hurdles to overcome before it can be widely used as a bioink, despite its promising qualities. Consistent bioprinting performance is severely restricted by the batch-to-batch variability in natural TG sources, which restricts repeatability. Furthermore, it is difficult to adjust TG’S mechanical and bioactive properties without compromising cytocompatibility. Its translation from bench to bedside is further complicated by practical considerations such as sterilization techniques, large-scale production and compliance with clinical and regulatory standards. There are significant gaps in our knowledge of TG-based bioinks. Rational bioink design is hindered by the limited systematic research that correlates rheological parameters with cell viability, printing resolution and post-printing stability. Furthermore, the long-term effects of biofunctionalization, composite formulations, and covalent alterations on tissue integration and performance remain largely unexplored. There is also limited data on how biological outcomes are affected by enzymatic crosslinking techniques, and how TG interacts with other biomaterials in composite systems. A multifaceted strategy will be needed to address these challenges. Reproducibility will improve through the physicochemical characterization and standardization of TG sources. Optimal printing conditions will be determined by systematically mapping rheology against printing resolution and cell viability through processing performance studies. The development of safer, cytocompatible chemical modifications and study of composite inks such as TG combined with cellulose nanocrystals, proteins or bioactive peptides can improve functional versatility. Finally, in order to facilitate clinical translation, efforts should focus on scaling up production, optimizing sterilization procedures, and navigating regulatory pathways. Collectively, these strategies will position TG as a reliable, adaptable and sustainable bioink for advanced tissue engineering and regenerative medicine applications.

Despite TG’s favorable printability and cytocompatibility, several critical issues must be addressed to fully unlock its potential in translational tissue engineering and advanced biofabrication. Material standardization and batch variability is one of the primary challenges, as TG is a naturally derived polysaccharide with heterogeneity in its composition depending on the plant origin and extraction methodology. Future research should emphasize the systematic physicochemical characterization of TG, including molecular weight distribution and polydispersity, to achieve reproducible rheological and biological behavior, similar to standardization efforts with gelatin and alginate derivatives. Further TG-based bioinks will depend on the development of advanced and controllable crosslinking strategies, while existing TG formulations rely on ionic crosslinking or physical interactions, incorporating photo-responsive or enzyme-mediated crosslinking strategies. It could offer enhanced control over degradation rates and mechanical properties, better matching the dynamic microenvironment of native tissues. The bioactivity of TG matrices represents another promising research direction. Functional modification of TG with cell adhesive peptides, extracellular matrix, or growth factors has the potential to improve cellular attachment, migration, and lineage-specific differentiation, particularly for applications where TG’s inherent bioinert nature limits early cell interactions. From a fabrication perspective, future studies should focus on multimaterial and gradient bioprinting strategies, where TG-based bioinks combined with complementary polymers may be used to create constructs with spatially controlled mechanical and biological properties. Such strategies are mimicking complex tissue architectures such as osteochondral and vascularized systems. Finally, translation toward clinical and industrial applications remains a major hurdle. Comprehensive long-term in vivo evaluations, immunological safety assessments and regulatory considerations must be systematically addressed. Moreover, scaling TG-based bioinks with automated, high-throughput bioprinting systems and good manufacturing practice standards will be crucial for their successful clinical adoption.

## 8. Conclusions

TG has significant promise as a bioink component owing to its intrinsic biocompatibility, adjustable rheological behavior and potential to support cell viability and tissue development. Its ability to form hydrogels and shear-thinning behavior makes it a suitable candidate for extrusion-based 3D bioprinting. Despite these benefits, there are some technical obstacles to overcome before TG can be widely adopted in mainstream bioprinting—the natural sources of batch-to-batch variability, inconsistent chemical changes, and lack of knowledge regarding its long-term mechanical and biological performance. A well-defined, clearly structured roadmap is required to advance TG-based bioinks towards clinical and industrial applications. The development of standardized and reproducible TG derivatives, integration with other functional biomaterials, systematic mapping of rheological properties against cell viability, and through in vivo research to verify its safety and functionality are the key priorities. By addressing these challenges, TG will position itself as a versatile and sustainable bioink platform, creating new opportunities for tissue engineering and regenerative medicine.

## Figures and Tables

**Table 1:** Comparison of tragacanth gum with alginate, gelatin/gelma, and pectin on the basis of key metrics.

Metric(s)	Tragacanth Gum (TG)	Alginate (Sodium Alginate)	Gelatin/GelMA	Pectin	References
Shear thinning behavior	Strong shear thinning, flow index generally low (~0.15–0.5)	Good shear thinning; tunability (~0.2–0.6) depends on molecular weight and concentration	Shear-thinning when blended with gelatin or fillers, usually ~0.25–0.8 influenced by temperature and polymer concentration	Moderate shear-thinning, lower than alginate unless reinforced; some cases reported *n*~0.3–0.7	[22,71,72,73]
Yield stress (Pa)	Moderate to high (~20–200 Pa), increase with particulate reinforcement	Broad range (~10–300 Pa) depending on concentration (2–8 wt%)	Generally, ~5–150 Pa yield stress; stability improved with fast cooling or photogelation	Low to moderate yield stress (~5–100 Pa); reinforcement often needs requirement	[22,72,73,74]
Printability	Good when blended; pure TG often swells excessively	Excellent filament shape and stacking when Ca^2+^ crosslinking is used	Very good with rapid photoinitiated crosslinking (or gelatin with temperature control).	Moderate for single-component systems: improved with crosslinkers/fillers	[22,72,73,74]
Crosslinking mechanism	Requires chemical modification or mixing with ionic polymers such as alginate + Ca^2+^	Immediate ionic gelation with Ca^2+^	GelMA: rapid photo crosslinking; gelatin alone offers thermo-reversible gelation on cooling	Low-methoxyl pectin cosslinks with ionic (Ca^2+^); chemical and enzymatic methods used for stronger gels	[20,73,74,75]
Compressive modulus (kPa)	TG gels often weak (≈1–5 kPa); reinforced/modified show much higher moduli around 20–50 kPa	Widely tunable: alginate hydrogels (2–8 wt%) often show compressive a modulus in the range ~1–100 kPa depending on concentration and crosslink density	GelMA hydrogels span soft to relatively stiff, depending on formulation; compressive modulus range ~0.5–50 kPa	~0.5–30 kPa; reinforcement with nanocellulose or other fillers	[22,73,74,75]
Degradation	TG is biodegradable, non-toxic; takes weeks, faster unless chemically crosslinked	Slow degradation due to lack of mammalian alginate lyase	Faster under physiological conditions (enzymatic/hydrolytic); days to weeks unless strongly crosslinked or chemically stabilized	Presence of pectinases and suitable pH can speed up hydrolysis	[20,74,75,76]
Cell viability	~70–90% in blends; but limited long-term studies	>80% but requires bioactive additives for adhesion	~80–95% viability; supports cell spreading, adhesion, and differentiation.	>70–90% viability, need added adhesion motifs (e.g., RGD peptides, proteins) for cell attachment/spreading.	[20,73,74,75]
Bioactivity (cell adhesion)	Low; needs ECM proteins/peptides	Low; requires modification (e.g., RGD conjugation) or blends	Cell-adhesion sites (from collagen/gelatin origin), enabling good cell attachment, spreading, and signaling	Low to moderate; improved with proteins/peptides	[20,73,74,75]
Sterilization	Gum is relatively heat-stable; TG blends may be sterilized by filtration or gamma irradiation	Alginate solutions can be sterile-filtered (especially low–MW) or autoclaved, and ionic crosslinking (Ca^2+^) must be controlled	Temperature-sensitive; sterilized by filtration; for photocrosslinking, photoinitiators and light dose must be optimized to avoid cytotoxicity	Sterilized by autoclaving or sterile filtration; modified pectins require purification	[20,23,74,75,76]
Cost and availability	Moderate; less standardized for biomedical use	Inexpensive; widely stocked in labs	GelMA requires synthesis or can be purchased; cost is moderate and predictable	Low to moderate cost; research-grade available from several suppliers.	[20,23,71,74]

**Table 2:** Research on tragacanth-gum-based bioinks/hydrogel scaffolds for extrusion-based 3D bioprinting.

Sr. No.	TG Composition/Concentration	Purpose of the Study	Crosslinking/Stabilization	3D Printed Method/Parameters	Biological Evaluation	Findings	References
1.	TG-based scaffold with different wt% formulations	To optimize TG concentration for extrusion printability, shear-thinning behavior, and post-printing shape fidelity.	Stabilization through physical enlargement and polymer interactions	Extrusion-based scaffolds; rheology-based optimization	Cell compatibility testing	Optimized TG blends demonstrated high shear-thinning and rapid recovery to excellent structural fidelity post-printing.	[89]
2.	TG incorporated with CNC to form polysaccharide nanocellulose composite	To enhance the mechanical strength and pore retention of TG-based scaffolds using CNC while maintaining cytocompatibility.	Non-covalent physical crosslinking supported by hydrogen bonding	DIW followed by freeze-drying to obtain a porous scaffold	Fibroblast survival and attachment assays	CNC raised elastic modulus and yield stress while retaining shear-thinning to improve pore retention and high cytocompatibility.	[22]
3.	Multi-component hydrogels containing TG, polysaccharides, nanoparticles	To evaluate TG as a viscosity modifier and mechanical stabilizer in multi-component composite bioinks.	Varies from physical to ionic crosslinking depending on composition	Extrusion printing from selected formulations	In vitro cytocompatibility	TG increases zero-shear viscosity and enhances post-printing mechanical integrity.	[58]
4.	Broad TG blends with alginate, gelatin, CNC, HA, chitosan	To summarize the role of TG as a rheology and printability enhancer across diverse bioink systems.	Summarizes ionic, physical, and covalent crosslinking strategies	Summary of outcomes from extrusion and hybrid bioprinting	-	Concludes that TG (≈0.5–2 wt%) is widely used as a rheology/printability enhancer in bioinks.	[20]
5.	Alginate (≈3 wt%) + TG (≈1 wt%) with or without hydroxyapatite	To improve filament fidelity and osteogenic bioactivity in alginate-based bioinks through TG and HA incorporation.	Ca^2+^ ionic crosslinking for gelation	Extrusion bioprinting with optimized pressure for smooth filament deposition	Cell viability and osteogenic assays	TG addition increased yield stress and improved filament fidelity; HA improved bioactivity and supported mineralization.	[69]
6.	KGM blended with TG at various ratios	To investigate the effect of TG on thermo-responsive gel strength for potential temperature-assisted bioprinting.	Thermo-responsive gelation	-	-	TG altered gel strength and storage modulus, indicating potential for temperature-assisted bioprinting systems.	[90]
7.	Pure TG and blends with gelatin/starch (0.1–5 wt%)	To establish baseline rheological properties of pure TG and TG blends relevant to extrusion bioprinting.	Physical gelation	Used to establish the baseline rheology relevant for printing applications	-	TG showed strong shear-thinning and thixotropic recovery even at low%, supporting its suitability for bioprinting formulation.	[91]
8.	PCL backbone combined with TG hydrogel and bioactive glass with or without CNC	To design hybrid constructs that combine mechanical support (PCL) with bioactive TG-based hydrogels for bone tissue engineering.	Hybrid: PCL as a solid scaffold with TG physically entrapped	Dual-material 3D printing (PCL melt extrusion + hydrogel deposition)	Osteogenic cell studies and mineral formation tests	Multiphase construct improved cell infiltration and mineral-like tissue deposition compared with PCL alone.	[92]
9.	Oxidized TG derivatives via periodate treatment	To develop chemically crosslinkable TG derivatives with tunable mechanics suitable for robust bioink formulations.	Chemical crosslinking through aldehyde-based reactions	Formation of chemically crosslinked hydrogel networks suitable for printing	Cytocompatibility, physicochemical analysis	Oxidation tailored crosslinking density and mechanics, offering roadmap toward stronger covalently crosslinked TG bioinks.	[93]

## Data Availability

No new data were created or analysed in this study. Data sharing is not applicable to this article.

## Abbreviations

The following abbreviations are used in this manuscript:

Abbreviation	Full Form
3D	Three-dimensional
TG/GT	Tragacanth gum/gum tragacanth
CMT	Carboxymethyl tragacanth
GelMA	Gelatin methacryloyl
PVA	Polyvinyl alcohol
PEG	Polyethylene glycol
CNC	Cellulose nanocrystals
HA	Hydroxyapatite
PEMA	Pectin methacrylate
PAM	Polyacrylamide
MTT	3-(4,5-dimethylthiazol-2-yl)-2,5-diphenyltetrazolium bromide
ECM	Extracellular matrix
UV	Ultraviolet
DIW	Direct ink writing
HRP	Horseradish peroxidase
H_2_O_2_	Hydrogen peroxide
PCL	Polycaprolactone
RGD	Arginine-glycine-aspartic acid peptide motif
DI	Deionized
